# Interactive Language Learning by Robots: The Transition from Babbling to Word Forms

**DOI:** 10.1371/journal.pone.0038236

**Published:** 2012-06-13

**Authors:** Caroline Lyon, Chrystopher L. Nehaniv, Joe Saunders

**Affiliations:** Adaptive Systems Research Group, University of Hertfordshire, Hertfordshire, United Kingdom; Indiana University, United States of America

## Abstract

The advent of humanoid robots has enabled a new approach to investigating the acquisition of language, and we report on the development of robots able to acquire rudimentary linguistic skills. Our work focuses on early stages analogous to some characteristics of a human child of about 6 to 14 months, the transition from babbling to first word forms. We investigate one mechanism among many that may contribute to this process, a key factor being the sensitivity of learners to the statistical distribution of linguistic elements. As well as being necessary for learning word meanings, the acquisition of anchor word forms facilitates the segmentation of an acoustic stream through other mechanisms. In our experiments some salient one-syllable word forms are learnt by a humanoid robot in real-time interactions with naive participants. Words emerge from random syllabic babble through a learning process based on a dialogue between the robot and the human participant, whose speech is perceived by the robot as a stream of phonemes. Numerous ways of representing the speech as syllabic segments are possible. Furthermore, the pronunciation of many words in spontaneous speech is variable. However, in line with research elsewhere, we observe that salient content words are more likely than function words to have consistent canonical representations; thus their relative frequency increases, as does their influence on the learner. Variable pronunciation may contribute to early word form acquisition. The importance of contingent interaction in real-time between teacher and learner is reflected by a reinforcement process, with variable success. The examination of individual cases may be more informative than group results. Nevertheless, word forms are usually produced by the robot after a few minutes of dialogue, employing a simple, real-time, frequency dependent mechanism. This work shows the potential of human-robot interaction systems in studies of the dynamics of early language acquisition.

## Introduction

The advent of humanoid robots has enabled a new approach to investigating the acquisition of language, and in this article we report on the development of robots able to acquire linguistic skills. Our work focuses on early stages analogous to some characteristics of a human child of about 6 to 14 months, the transition from babbling to first word forms, a critical stage in the development of linguistic skills [1]. No knowledge of segmentation into words or syllables is assumed. As well as being necessary for learning word meanings, the acquisition of anchor word forms facilitates the segmentation of an acoustic stream through other mechanisms. The work described here is conducted through the ITALK project [2], which elsewhere includes research into the concomitant acquisition of referential meaning and syntax [3].

We take the position that numerous factors contribute to language acquisition but it can be worthwhile to examine these separately. We investigate one mechanism among many that may contribute to the acquisition of word forms, a key factor being the sensitivity of the learner to the statistical distribution of linguistic elements. We show how word forms can be acquired, assessing the extent to which our model presents a plausible analogy to human linguistic development, and how it diverges. An apparent problem with variable pronounciation may in fact aid word form acquisition.

The learning of word forms is concomitant with, or possibly a prerequisite for, learning word meanings. “The detection and exploitation of […] statistical properties of ambient speech thus allows infants to find candidates in running speech before they know the meanings of words” [4] (page 137). Learnt word forms may then come to be associated with particular objects or events [5], [6].

Furthermore, whether or not the meaning is known, isolated word forms contribute to the segmentation of an acoustic stream of sound into discrete components [7]–[9].

Our approach accords with recent neuroscientific research and developmental psychology which indicate that dual systems are needed for language processing. Ventral pathways are concerned with relating sounds to meaning, while the dorsal pathway is involved with relating sounds to articulatory productions, detecting phonological patterns and word forms [10], [11]. In the work described in this paper we investigate processes analogous to some in the dorsal pathway alone.

A critical component of early human language learning is *contingent* interaction with carers [12]–[17]. Therefore we have conducted experiments, described here, in which human participants interact with the humanoid iCub robot [2], aiming to teach it some word forms. (In this article the terms “participant” and “teacher” are used interchangeably.) We show how word forms may be learnt through a dialogue, in which naive participants talk naturally, and find some of the characteristics of child directed speech (CDS) or motherese in their talk to the child-like humanoid robot. Initially, the robot babbles random syllables, but as the interaction progresses its productions become biased towards the teacher’s speech, which is a step on the way to learning single-syllable word forms. The algorithm is described below. The syllabic structure of possible English syllables is presupposed in our system. A corpus of sentences which provide almost total coverage of permissible demi-syllables in English (which combine to make syllables) can be found in [18].

The speech of the participant is perceived by the robot as a stream of phonemes, not segmented into syllables or words, which leads to numerous possible ways of representing the speech as syllabic segments. In addition the pronunciation of many words in spontaneous running speech is variable. Phonetic variability of function words is comparatively high, and so the corresponding phoneme sequence may not be stable across occurrences. However, in line with research elsewhere that has influenced our work [19], we observe that salient content words often emerge among the more frequent syllables with consistent phonemic representation and their frequency will have an effect on the robot’s talk.

The robot’s output is syllabic. Since it is not in general possible to produce consonant phonemes in isolation (apart from a few exceptions such as *shh*) syllables must normally contain a vowel. This syllabic basis is not inconsistent with the key role played by phonemes, for instance in distinguishing minimal pairs - similar words that differ in one sound, such as *dog* and *fog*. As has long been understood, phonemes themselves are abstractions from the acoustic signal; there is no invariant mapping of acoustic cues to phonemes as the realization of a phoneme depends on its context [20], [21]. Phonemic signatures are hard to identify in the acoustic stream by automated processes: how humans do this is an active area of research [22],[23], while practical applications sidestep the problem with ingenious engineering approximations [24].

In contrast to this infants in their first few months can distinguish different phonemes, even before they can produce them. Examples of work in this field include [22], [25]–[27].

Our approach is based on observations that in human infants there is a close connection between perception and production of speech sounds, one of several facets of language learning. The neural mechanisms that effect this connection are widely debated [28], but infants learn the sounds of their own ambient language, and practice what they hear (see, for instance, [29]–[31]). Children born profoundly deaf cannot learn to speak normally. The typical production of syllabic babble has been reported from extensive observations of many children, and the practice of these sounds primes the same neurons that will engage with the perception of such syllables, if they are within repertoire. There is an auditory-articulatory loop [32]. However, while motor involvement in speech production is critical, in speech perception it is not essential, though often observed [33].

### Word Form Acquisition and Segmentation

As well as being a stage in the process of understanding the meaning of words, word form acquisition contributes to the task of segmenting the acoustic stream of speech into syllables and words. A number of mechanisms are involved in segmentation, including factors relating to prosody, sonority, utterance length, temporal structure, distributional statistics and phonotactic constraints, and these mechanisms produce candidate segments. Now, given a string of phonemes to be segmented into syllables, the number of possible candidate partitions increases exponentially with length. If the length is restricted by occurrences of known anchor items this can make a significant contribution [7]–[9]. Such words may have been heard as isolates [34] or may have been acquired through a process analogous to that displayed in our experiments.

### Methodology in Context

Interest in child language acquisition goes back to the earliest recorded times. The ancient Greek historian Herodotus, circa 450 BC [35], writes about an experiment in which two infants were shut up alone together, fed by a shepherd who was ordered never to talk to them, to see what words they would produce. They eventually came up with the word “bekos” for food. There are also other accounts in later times of cruel experiments with children deprived of human contact.

Many records of child language acquisition were produced in the 19th century, based on diaries reporting the development of single children, Clark [36] (page 15) gives a list. In the 20th century quantitative as well as qualitative approaches were adopted. Gesell researched the stages in child development through systematic observation of large numbers of children. Piaget’s theories drew on studies of small groups of children, Vygotsky observed the crucial role of social interaction, and Skinner’s work on behaviourism was very influential.

In contrast to these empirical approaches Chomsky’s rationalist theories came to play a prominent role in the second half of the 20th century. At the core of his theories was his view that an innate universal grammatical faculty underpinned all human languages, and the task of researchers was to uncover this universal grammar. “ ‘Knowledge of language’ involves in the first place knowledge of grammar - indeed, […] language is a derivative and perhaps not very interesting concept” he wrote [37] (page 90).

Towards the end of the 20th century empiricism reasserted itself. A corpus based approach was widely undertaken, involving the shallow examination of large quantities of data in contrast to a deep analysis of small samples of language. Increasing computer power made large scale analysis feasible, and other technical advances associated with an empirical approach, such as the development of information theory and neural computing, produced promising results. Meanwhile the rationalist search for that alluring goal, an underlying core grammar, seemed ever more elusive.

### Recent Developments

In recent decades two significant changes have altered the landscape. The development of neuroscientific investigative techniques has enabled some theories of language processing to be subjected to empirical tests. For instance, Chomsky’s idea that a Language Acquisition Device might have a specific location in the brain has not been substantiated. Instead, language processing has been shown to be distributed. Chomsky himself has said that “the faculty of language” is “more or less on a par with the systems of mammalian vision” [38] (page 2). Other results of neuroscientific research have illuminated the acquisition and processing of language - one example that significantly influences our work is evidence for dual processing pathways [10], [11].

The second development is the potential to investigate language acquisition through computer simulations and experiments with robots. Steels undertook pioneering work in modelling the evolution of communication between embodied software agents, such as his “Talking Heads” experiments [39].

These two factors come together in models and simulations of neural processes. An influential model of speech acquisition and production has been developed by Guenther, Ghosh and Tourville [40]. This focuses on the sensorimotor transformations underlying the control of articulator movements, taking as input a speech sound string and outputting articulatory commands to a simulated vocal tract. Their work shows how an initial auditory target prompts the production of a speech sound through a sequence of feed forward commands and feed back controls. To instantiate the target, a syllable, word or short phrase is presented to the model by a human speaker: “[the] model is given a phoneme string by the modeler, and the model produces this string in the specified order” (*ibid*, page 294). The human teacher presenting the model with an isolated word contrasts with with our own system in which the teacher produces spontaneous, unscripted continuous speech in a proto-conversation with the robot. Salient words can be learnt without restricting the human to a prescribed isolated word.

Our work takes a similar approach to that of Breazeal, in which robots interact with humans providing a variety of social cues to support engagement via natural, personal interactions [41]. As in her work, we study interactions with naive participants, in contrast to experiments using people with technical expertise. In the linguistic field a similar approach is adopted by Steels and Kaplan [42], in which the robot Aibo learns the meaning of words through social interaction. It differs in that word forms are assumed known (*ibid*, page 18), whereas our work focuses on the preliminary stage of learning word forms. Like them, our goal is to examine specific issues on the emergence of communication, and one advantage is that we can analyse and extract data from internal states in the learning process. In our case this is described below in the Results section.

We take a constructivist approach to language learning, as described by Tomasello [43]. Though the work reported here focuses on preliminary word form learning, this approach has also inspired research into semantic language learning, grounding words with objects through audio, visual, proprioceptive and spatial cues, e.g. [3],[44]–[47]. Computational models of the acquisition of linguistic competencies includes Oudeyer’s work on categorical perception, demonstrating the development of phonemic categories through self-organization [48]. Research in this field also includes work with populations of interacting synthetic agents, for instance the acquisition of vowel systems modelled by de Boer [49], extended to syllables by Oudeyer [50]. A functional model which aims to integrate different phenomena involved in phonological processing and word form learning has been developed by Gupta and Tisdale [51].

Since our work concerns the acquisition of a human language by a robot we are inspired by the process in humans. Thus the basis of our experimental work is a real-time interactive situation where a human participant talks to a robot, using his or her own spontaneous words. We identify some of the key processes that can be observed, and though these processes are typically interlinked in complex networks of associations, for the purpose of our research we initially look at them independently to see what contribution different mechanisms can make.

It is worth stressing that synthetic language, such as robot to robot communication, is fundamentally different from human language, which comes with its accumulated evolutionary baggage, and exaptations of primitive processes [52]. For example consider how a “logical” language in which each phonetic string of sounds would map onto one and only one meaning [53] contrasts with the observed frequency of homophones in English, French, Chinese, Japanese and other languages, possibly all (“one, won”, “two, to, too” etc.). In English in a corpus of about 1 million words, 20 of the 50 most frequently occurring words are homophones [54]. We usually have no difficulty in disambiguating them by taking short sequential contexts, re-using primitive sequential processing mechanisms.

From extensive psycholinguistic research we take as a premise the observation that infants are sensitive to the distributional frequencies of the sounds they hear in speech directed towards them [25], [26]. Our work, focusing on analogies with infants aged about 6 to 14 months, models possible mechanisms contributing to the transition from babbling to first words. We propose methods by which the robot might perceive and produce syllabic output, and by analysing the spontaneous speech of the participant teachers we see how the robot might learn emerging salient words.

## Methods

### Ethics Statement

This research was approved by The University of Hertfordshire Ethics Committee for Studies Involving Human Participants. Informed consent was obtained in writing from all participants.

### Scenario - Dialogue with Robot DeeChee

As the purpose of these experiments is to investigate word form learning through interaction with a human teacher, it is critical for the robot to elicit an appropriate approach in the teacher, and therefore it is important that our system appears to be embodied in a real robot, rather than a software agent. We take our robot to have neutral gender but participants seemed to consider it was a boy.

**Figure 1:**
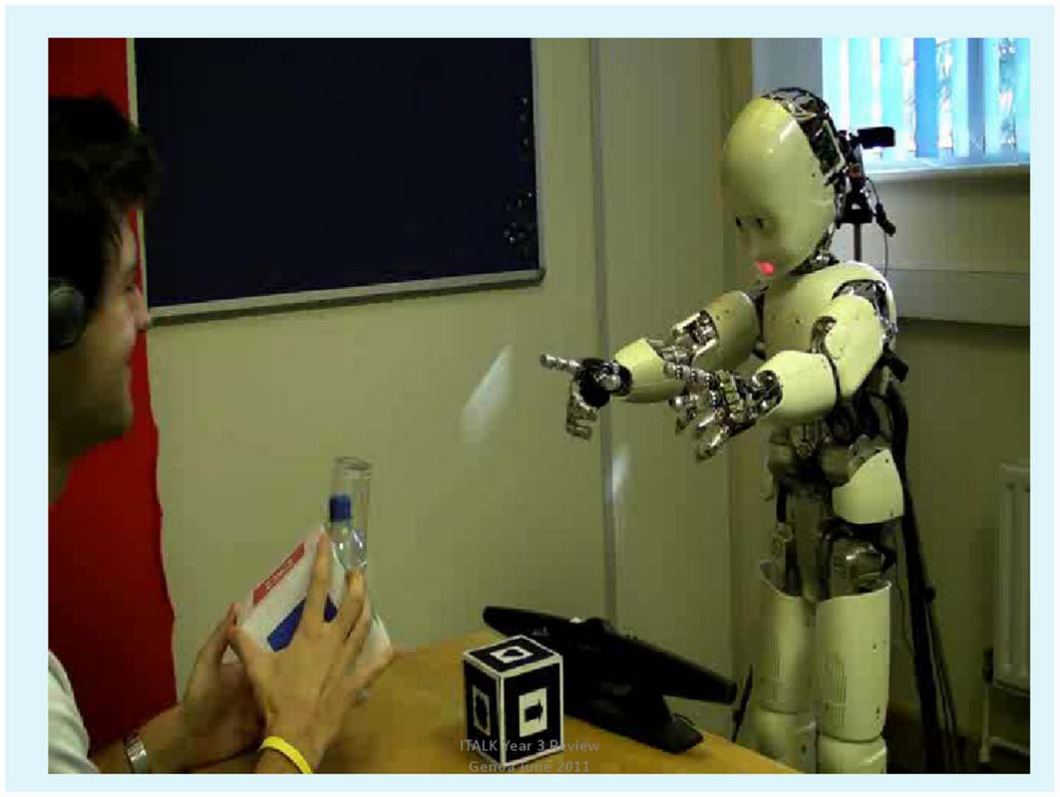
The scenario for the human-robot dialogue.

The experimental scenario is a real-time, on-line dialogue between a human “teacher” and the small humanoid iCub robot named DeeChee, as shown in Figure 1 and in the video clip in Video S1. The video clip can also be seen at http://youtu.be/eLQnTrX0hDM (note that the ‘0’ is zero).

On a table between them are some blocks of different colours with various shapes on them. The participant is asked to talk to DeeChee, using his or her own words, as if it was a small child, and to try to teach it the names of colours and patterns. It so happens, as shown in Table 1, that in this scenario nearly all the salient words, 20 out of 24, have one syllable, and are of the form 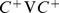, where C is a consonant and V is a vowel. The notation 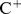 means one or more instances of C. For convenience in this paper we represent 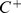 by C, and investigate the learning of CVC word forms.

**Table 1:** List of salient words used by participants.

One syllable words of form CVC	Other salient words
big	arrow
black	blue
box	circle
cross	crescent
cube	
green	
heart	
moon	
red	
ring	
round	
shape	
shapes	
small	
smile	
square	
squares	
star	
sun	
white	

Salient content words which were spoken by participants in these experiments. Recall that in our notation C represents one or more instances of a consonant.

The participating “teachers” are volunteers not involved with the project. Five sets of experiments were conducted with 34 participants, who were varied in age, occupation, gender, experience of children and familiarity with computers. Their spontaneous speech ranged from the extremely loquacious to the quite inarticulate. They were paid £5 in recognition of their help. Most were either university administrative staff or PhD students from other disciplines.

There were 7 different participants in each of sets 1,2, 4 and 5, and 6 in set 3. In sets 1, 3, 4, 5 conditions only varied in the humanâ® “robot interface, while set 2 was based on a variation in the learning algorithm, described later. Reference is also made to 2 preliminary sets of experiments: one with 8 participants talking to another robot in a similar scenario but then processed off line [55]; the second with 2 participants in a real-time interaction but with a simulated reinforcement mechanism [56].

### Experimental Programme

The 5 sets of experiments described here were conducted in sequence. Due to the embodied and situated nature of the scenario we followed an iterative approach to interaction design, aiming to improve performance in terms of learning word forms.

A feature of all the experiments was that teachers had to listen to DeeChee’s babble and take notice of any words. With set 1 it was found that teachers often missed hearing words uttered by DeeChee in among the babble and thus did not give reinforcement. The program was adapted for set 2 by introducing a filter, designed to facilitate the teachers task of identifying words in a stream of babble. On the supposition that the task would be easier if there were less syllables to chose from, a filter was introduced, so the robot had a smaller syllabic vocabulary. This filter, the syllabifier, was trained on a corpus of known words, and aimed to identify and exclude syllables that were unlikely to be words. However, it filtered out a large number of syllables that were candidate words and was not used for the subsequent sets. Sets 3, 4 and 5 reverted to the original program, but we had the intention of lowering the cognitive load on teachers, and thus hopefully increasing their word recognition rate, by progressively simplifying the guidelines each time. As examples the guidelines for set 1 and for set 5 are in Appendix S1.

In all experiments participants were asked to try to teach DeeChee the names of shapes and colours, and to take turns speaking. They were asked to talk with DeeChee as if it was a small child, to listen to its babble and make an approving comment if it uttered a proper word form. In set 1 and 2 teachers were told to talk when DeeChee smiled, pause when DeeChee blinked and stopped smiling. However, it seemed that the task of listening to the babble needed a significant degree of concentration, as a number of correct word forms were missed, and watching DeeChee’s facial expression was a distraction. For sets 3, 4 and 5 the guidelines were simplified, on the assumption that too heavy a cognitive load might contribute to the low level of teacher response when DeeChee uttered correct word forms. Explicit description of the facial expressions were omitted from the guidelines, though they remained as before, as implicit support for turn taking. Also in these sets the need to listen carefully was emphasized.

The different guidelines were:

Set 3: instructions on procedure without an explicit instruction to make an approving comment if DeeChee uttered a proper word.

Set 4: instructions on procedure similar to previous, but with explicit instruction to make an approving comment.

Set 5: instruction on procedure reduced to a single request to listen to DeeChee, and make an approving comment if appropriate. Other instructions, such as on the use of the microphone etc. were moved to introductory material.

### Hypotheses

We hypothesize that.

1. A synthetic agent embodied in a humanoid robot can learn one-syllable word forms through interaction with a human teacher talking naturally;
2. Word form learning is augmented by contingent reinforcement, if the teacher makes an approving comment when a proper salient word form is uttered.

### Algorithm

Robot DeeChee perceives the teacher’s speech as a stream of phonemes, not segmented into syllables. An overview of the algorithm is shown below. An example of an unsegmented stream of phonemes, using letters as pseudo-phonemes, would be “a r e d b o x”. The set of all possible syllables (each of which must include a vowel) would be *a, ar, re, red, e, ed, bo, box, o, ox*, which assumes no syllable segmentation knowledge. A “real word form” is any proper word, not necessarily one with the right meaning in a given context, a “salient” word is an information carrying word that the participant is trying to teach.

Initial state: DeeChee produces random syllabic babble

Repeat until dialogue time ends:

  utterance T : Teacher speaks, speech represented by a stream of phonemes

  process : DeeChee perceives input as set of all possible syllables from stream,

       frequency table for each of these syllables is incremented

  utterance D : DeeChee produces quasi-random babble, biased to teachers input

  process : Teacher listens to babble, to hear for a real salient word form

       if teacher hears any salient real word form

          then teacher reinforces

  process : if DeeChee perceives reinforcement

       then previous utterance is analysed

          word is selected by heuristic and stored in its lexicon

At the start of each experiment DeeChee produces random syllabic babble. It can turn its head and change its facial expression minimally, smiling and blinking. Its arms can move towards or away from the blocks which are being shown by the teacher.

### Assumptions

The following assumptions are made:

- DeeChee practices turn taking in a proto-conversation
- It can perceive phonemes, analogous to human infants
- It is sensitive to the statistical distribution of phonemes, analogous to human infants
- It can produce syllabic babble, but without the articulatory constraints of human infants, so unlike a human of this age it can produce consonant clusters
- It has the intention to communicate so reacts positively to reinforcement, such as approving comments

Real-time reinforcement is based on the teacher uttering approving comments, such as “well done”, “good”, “clever”, etc. When DeeChee recognizes one of these terms then a one-syllable word from its previous utterance is saved in its lexicon. Now, DeeChee’s previous utterance will be multi-syllable, and the appropriate part must be identified. This is done using a heuristic, based on frequency, recency of use by the teacher and type of syllable. The heuristic produces a score calculated for each syllable, and the CVC syllable with the highest score is reinforced, (recall that in our notation ‘C’ represents one or more instances of a consonant) where


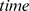
 = elapsed time since syllable was last uttered by teacher
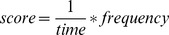



The approving comments themselves, against which DeeChee’s perception is matched, were taken from preliminary experiments [55].

### Method of Investigation

Each of the experiments consists of 2 consecutive 4-minute dialogue sessions between a teacher and DeeChee, giving the participant a break in the middle. For each participant learning was carried forward from the first to second session. Learning was separate for each participant and started anew each time. The 4 minute session length was chosen after some preliminary trials, since with longer sessions attention flagged with some participants. We noted that some critical experiments with human infants, on learning to detect phonemic patterns, were conducted for only 2 minutes [25].

Initially DeeChee produces random syllabic babble, then the teacher speaks, and the turn taking continues. The phonemic alphabet used is the CMU phoneme set [57], as shown in Table 2. The syllabic babble that DeeChee produces is of the form V, CV, VC or CVC where V is a vowel and C is either a single consonant or a cluster of consonants. Thus, in our notation syllables such as *square* and *box*, (*s k w eh r*) and (*b aa k s*), are denoted by the form CVC rather than 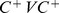. Almost all allowable English combinations are possible, as described in the SCRIBE corpus [18], with clusters of up to 3 consonants. (Clusters of more than three consonants are excluded, such as in *glimpsed.*) “Allowable” means clusters that occur in the ambient language. Some clusters can only occur at the beginning of a syllable, such as (*g r*) as in *green*, some only at the end, such as (*k s*) as in *box*, some at either position, such as (*s t*) in *star* and *last*.

**Table 2:** The CMU phoneme set.

Phoneme	Example	Phoneme	Example
aa	odd	k	key
ae	at	l	lee
ah	hut	m	me
ao	ought	n	knee
aw	cow	ng	ping
ay	hide	ow	oat
b	be	oy	toy
ch	cheese	p	pee
d	dee	r	read
dh	thee	s	sea
eh	Ed	sh	she
er	hurt	t	tea
ey	ate	th	theta
f	fee	uh	hood
g	green	uw	two
hh	he	v	vee
ih	it	w	we
iy	eat	y	yield
jh	gee	z	zee
		zh	vision

The teacher’s speech is converted to a stream of phonemes, using an adapted version of Microsoft SAPI 5.4 [56]. It is perceived by DeeChee as a stream of phonemes, with consonant clusters found. All possible syllables are extracted as the teacher’s phoneme stream is expressed, and stored by DeeChee in frequency tables. Recall that these syllables, of the four types described above, will be overlapping as there is no segmentation knowledge.

Prior to the main experiments participants were trained for about 10 minutes on the speech recognizer, since an adapted version of this was used to represent the teacher’s speech as a stream of phonemes.

Turn taking is based on a timing mechanism for utterances: DeeChee babbles for 4 seconds then listens for 4 seconds before babbling again. A dynamic method would be more realistic, and this method sometimes produced an unforeseen problem discussed in Section Results below: some participants did not stick to their turn but talked over the start of DeeChee’s utterance. DeeChee has a neutral expression as it babbles but blinks as it stops and its expression changes to a smile when it starts listening. Participants completed a short questionnaire after the experiment and most often had the impression that they were interacting directly with the robot.

DeeChee’s babble, a sequence of syllables composed of V and C phonemes, is converted to an audible output using the eSpeak synthesizer [58].

DeeChee’s output is determined first by a random choice of one of the four syllable types. Then, as the syllable frequency counts increase, DeeChees babble, still quasi-random, becomes biased towards the teachers speech: syllables that have been frequently perceived are more likely to be produced. To explain the algorithm suppose 3 syllables of the chosen type have been perceived. If *syl1* occurs once, *syl2* occurs 3 times and *syl3* 6 times, then the chances of DeeChee producing these syllables are respectively 1/10, 3/10, 6/10. Any of these 3 syllables may be produced, but with varying probabilities. The random generator was adopted because we have no principled reason to adopt any other method.

Then, if the teacher hears DeeChee utter, by chance, a salient (single-syllable) word he/she *may* make an approving comment. The term “may” is used because the behaviour of the human participants is not determinate. DeeChee “may” then perceive this approving comment. Here the term “may” is used because the phoneme recognizer does not always detect the comment. However, once the approving comment is recognized the word form is then lodged in DeeChee’s lexicon. This is the reinforcement process. The dialogue continues with learnt word forms now occurring more often in the quasi-random babble. When the random selector initially selects a syllable type the word in the lexicon will be a candidate to be chosen, along with the four syllable types. Thus, if there are two words in the lexicon, there will be six candidate items. Once in the lexicon a word form has a higher chance of being produced.

**Figure 2:**
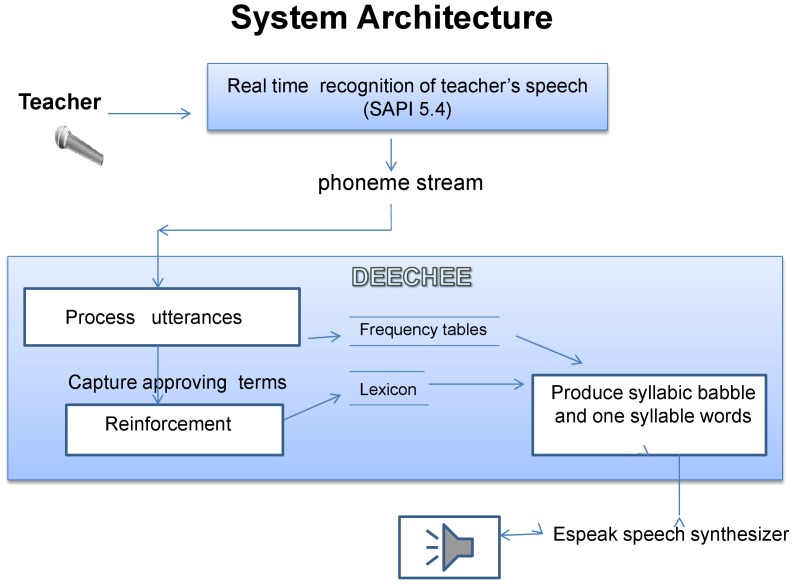
Overview of the system architecture.

An overview of the system architecture is shown in Figure 2.

In the preliminary experiments the reinforcement was simulated. The programmer made a list of salient words that it was hoped DeeChee would learn. If there was a match between one of these words and DeeChee’s output, then that word was entered in the lexicon [55], [56]. In contrast the reinforcement mechanism in the experiments described here depended on the teacher hearing a desired word uttered by DeeChee and responding with an approving comment. Then DeeChee should recognize this comment and select the appropriate one-syllable word from its utterance to be put in its lexicon, the selection of the appropriate syllable depending on the heuristic. In this work only CVC types are candidates for selection, because of the observed occurrence of syllable types, as shown in Table 1, and also for simplicity.

## Results

The questions which these experiments are designed to answer and results are summarised as follows:

1. As the dialogue progresses, does DeeChee’s babble begin to include some proper one-syllable word forms? Yes (Hypothesis 1).

2. (a) Does the teacher respond to the production of proper word forms? Sometimes.

(b) Does DeeChee recognize this reinforcement? Usually. (Hypothesis 2).

One example of a successful learning interaction is from participant 4A, and an excerpt extracted from her dialogue is shown below. The utterances from DeeChee and 4A are represented using CMU phonemes, as shown in Table 2. The term *ilex*, derived from “infant’s lexicon”, refers to DeeChee’s memory store of learnt syllables.

From individual example 4A

Brackets around DeeChee’s output show syllables

DeeChee: (ao ks) (ow dz) ae (r eh d) (ao s)

  4A: w eh l d ah n

    reinforcement term "well done"

    heuristic applied to previous utterance

    (r eh d) found, moves into ilex, DeeChee’s lexicon

contents of ilex: (r eh d)

…………

DeeChee: (iy n) (r ey n) (r ey n) (m ao dl) (kr ao s)

  4A: v eh r iy d g uh d eh n d

    contains reinforcement term "good"

    (kr ao s) found, moves into ilex

contents of ilex: (r eh d) (m ao dl) (r ey n) (gr iy n) (kr ao s)

3 words reinforced correctly: red, green, cross

3 non-words also reinforced by the end

The three non-words that were reinforced were derived from errors in the phonemic representation, coupled with over generous praise from 4A, as discussed further below.

Note that in these experiments we investigate the learning of CVC word forms, since these represent almost all the salient words in our scenario - see Table 1. The frequency of the other syllable types will be at least as high: for instance if *r eh d* occurs *n* times then *r eh* of form CV will occur *n* times or more, but this would not be reinforced.

### Detailed Analysis of Learning Interactions

Participants are shown the experimental set up (Figure 1) and asked to teach DeeChee the names of shapes and colours. Essentially, no restrictions on participant’s speech are given - they are left to use their own words; see Guidelines in Appendix S1. As the participants use their own words there may be a variety of terms for a shape, for instance *ring, round, circle*, or *moon, smile, crescent*. Then there are other non-salient proper words such as *this, that, look*. There are also non-words which may be learnt and reinforced in error. Some of these come from adjacent words run together such as *y uw s* from “can you see”. Other non-words come from a mismatch between the teacher’s utterance and its representation by the phonemic recognizer. Such mismatches are usually errors in the phonemic recognizer, but also may be idiosyncratic pronunciation. As the participants are asked to talk to DeeChee as if it is a small child, in some cases this results in excessive praise, whether DeeChee has produced a real word form or not. Thus non-words get reinforced.

In the following results we analyse both the interactive reinforcement, the actual real-time learning results in experiments with the participants, and also the “simulated reinforcement”. The latter is another way of interpreting data from these experiments which reflects what the robot would have learned if all the salient words that it uttered had been reinforced by the teacher. By uttering these words DeeChee showed that some learning had taken place, but the reinforcement step did not follow on. It is called “simulated reinforcement” since in preliminary experiments word forms uttered by DeeChee were compared to a list of salient words, and were treated as reinforced if there was a match.

As has been noted before, an advantage of this type of research method is that we can dissect a process and analyse internal states [42]. In this case we can break up the process into the following components of the interaction:

- The speech of the participants
- The perception of this speech by the robot as sets of all possible syllables in phonemic form
- The syllabic utterances of the robot and the production of candidate words
- The recognition by the participant of words in the robot’s babble followed by real-time reinforcement

### Speech of the Participants

Table 3 gives statistics for the participants’ speech. There is very marked variation among the participants, with the number of words spoken varying from 83 to 876. The number of different words used ranges from 7 to 145. The amount of repetition is indicated by the ratio of word count to the number of different words, and this ratio varies from nearly 4 to nearly 12. These figures show that experiments with naive participants must expect very varied performances.

**Table 3:** Statistics on participants’ speech, with robot’s perceptions and productions.

Col. 1	Col. 2	Col. 3	Col. 4	Col. 5	Col. 6	Col. 7	Col. 8
P’pant	Total no. of words from p’pant	No. of different words from p’pant	No. of different syllables perceived by robot	No. of different CVC syllables perceived by robot	No. of salient wordsin top 10 spokenCVC words	No. of salient sylls. intop 10 perceivedCVC sylls.	No. of salient words uttered by robot
Set 1							
1A	282	38	267	96	6	5	5
1B	387	76	358	126	4	0	2
1C	447	53	284	99	5	3	6
1D	481	78	317	116	7	5	4
1E	825	84	458	199	5	4	4
1F	559	121	453	189	5	3	1
1G	398	79	306	117	4	0	0
Set 2*							
2A	627	53	149	38	5	2	1
2B	475	61	98	21	5	2	2
2C	876	113	170	45	3	1	1
2D	832	113	176	44	4	2	0
2E	229	20	86	21	8	2	1
2F	729	109	151	28	1	0	1
2G	–	–	139	35	–	1	2
Set 3							
3A	454	57	316	114	4	3	4
3B	165	20	181	61	7	3	2
3C	330	56	297	112	8	4	2
3D	110	27	171	57	2	2	2
3E	297	48	304	118	7	3	3
3F	656	135	549	240	3	2	1
Set 4							
4A	611	92	426	180	4	4	3
4B	368	44	359	154	4	2	1
4C	654	84	515	225	6	1	2
4D	692	128	488	212	3	2	3
4E	704	100	476	203	4	2	2
4F	234	65	298	110	8	4	4
4G	180	24	227	80	5	3	3
Set 5							
5A	558	118	492	213	2	3	2
5B	681	145	539	235	5	3	1
5C	491	46	301	117	6	3	3
5D	221	53	243	85	4	3	2
5E	715	120	474	211	4	2	2
5F	189	20	139	46	6	5	5
5G	83	7	52	13	2	2	2
min	83	7	52	13	1	0	0
max	876	145	549	240	8	5	6
mean	470.9	72.3	344.1	138.1	4.7	2.8	2.6
SD	226.8	38.8	131	64.0	1.8	1.3	1.4

* See text, section “Experimental Programme” for description of Set 2. The min, max, mean and SD for columns 4, 5, 7 and 8 exclude Set 2, as the filtered figures are not comparable to those in other sets.

Pearson correlation between columns 6 and 7: 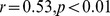;

between columns 7 and 8: 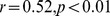, both excluding Set 2.

The speech used by participants, though very varied, typically had some of the characteristics of Child Directed Speech (CDS): short utterances, limited vocabulary, simple constructions, pronounced prosody, repetition [3], [4], [59], and, in some cases, praise of DeeChee’s speech regardless of its actual performance. It has been reported that humans speak to children and robots in different registers [60], but in these cases there was no user expectation that the robot was child-like. Whether the robot is simulated or embodied is also relevant. We found characteristics of CDS in other experiments with a humanoid robot in a similar scenario [3].

### Perception of Speech by the Robot

The speech from the participant is presented to DeeChee as a stream of phonemes, from which all possible syllables are formed and stored. The performance of phoneme recognizers is hard to assess; Greenberg reports that even with linguistically trained, highly experienced transcribers inter labeler agreement ranged from 80% to 72% on labelling 4 hours of spontaneous speech [19]. As we focus on one-syllable salient content words of the form CVC (see Table 1) we only looked at the recognition rates for these. Again, results were very varied. Taking the number of correct recognitions of the 3 most frequently spoken salient words for each participant the averages were 45% for set 3, 49% for set 4 and 61% for set 5.

There is a wide range in the total number of different syllables perceived by the robot for each participant, from 549 to 52, since they are derived from the variable input speech and variable levels of phoneme recognition. As well as the word counts for the teachers’ speech Table 3 shows the total number of different syllables, of the 4 types V, CV, CVC and VC, and the number of different CVC syllables perceived by the robot. The number of salient CVC items out of the top 10 most frequent for the transcribed speech and for the perceived syllables in phonemic form exhibit significant correlation (Pearson correlation 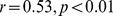).

Table 4 gives further data for set 1 as an example.

**Table 4:** Comparison of word counts with syllable counts as perceived by the robot: Set 1 as an example.

Participant	Number of different words spoken	Number of different CVC words spoken	Number of different CVC syllables as perceived by robot
1A	38	16	96
1B	76	50	126
1C	53	26	99
1D	78	36	116
1E	84	42	199
1F	121	55	189
1G	79	35	117

Note the difference in number of CVC words, when the speech stream is segmented into words, in contrast with the much larger number of syllables as perceived by the robot with no knowledge of syllable boundaries. This is in spite of the fact that some small part of the participant’s speech is not perceived when he/she talks over DeeChee out of turn.

Note that not all the participants’ speech is always perceived by the robot. The transcription of the participants’ speech is taken from the audio recordings, showing all that is spoken. However, at times the participant will talk over DeeChee instead of stopping at the end of his/her time-based turn, and DeeChee will then miss what is said.

In spite of the fact that there is no knowledge of word boundaries, and numerous candidate syllabic segments are generated, the salient one-syllable words are well represented in the top 10 most frequent syllables. This means they will probably influence DeeChee’s speech, who will thus be more likely to produce a word that will elicit reinforcement. We do not expect the frequent salient syllables to include all those that are spoken but hypothesize that enough are perceived to bootstrap the learning of some word forms.

There are three reasons for a single-syllable CVC word to lack a matching CVC syllable in phonemic form. Firstly, the speaker may not articulate the word in a canonical, dictionary form, as frequently happens in spontaneous running speech. Secondly, the words spoken may be outside the speaker’s turn, and thirdly the phoneme recognizer may not be operating effectively.

### The Robot’s Productions and Candidate Words

DeeChee produces syllabic babble that becomes biased towards the most frequently heard syllables from the teacher’s speech. Table 5 shows the results of analysing the number of salient one-syllable word forms in DeeChee’s output. We expect this to be correlated to the number of frequent salient CVC syllables, since in the quasi-random output more frequent syllables have a higher probability of being expressed (Pearson correlation 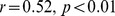).

**Table 5:** Words produced by the robot and those missed by the teacher for reinforcement.

Set number	Number ofparticipants	Salient words producedby the robot	Number of words missedby participants	Word reinforced but heurisitc failed (included in col. 4)
1	7	22	17	1
2	7	8	4	0
3	6	14	11	2
4	7	18	12	1
5	7	17	10	0
total	34	79	54	4

Aggregated figures.

Note the significant number of words uttered by DeeChee but not noticed by participant. “Simulated reinforcement” would find these matches.

Table 3 shows the relationship between the number of high ranking, frequent CVC syllables and the words produced by DeeChee. Note how many of these words are not noticed by the participants (Table 5).

The variable number of words produced depends partly on the vocabulary used by the teacher. In one case where DeeChee only output one proper word the teacher disregarded the guidelines on the “boring” task of teaching shapes and colours, and started giving a history lesson on the fall of Constantinople, with a vocabulary lacking the salient words in our scenario.

### Word Learning through Real Reinforcement

A summary of results is shown in Table 6. In set 1 a significant number of words uttered by robot DeeChee were not noticed by the teachers, not reinforced, and in successive sets of experiments we attempted to address this.

**Table 6:** Words learnt.

Set number	Number of participants	Salient words learnt	non-words learnt	Other words learnt
1	7	5	11	2
2	7	4	9	5
3	6	3	11	6
4	7	6	11	4
5	7	7	13	5
total	34	25	55	22

One-syllable words uttered by DeeChee, perceived by teacher, reinforced, and entered in lexicon as learnt. “Other words” are proper but non-salient words, such as “this” or “that”.

In set 2, in order to try and facilitate the teacher’s task of noting words in DeeChee’s babble a filtering process was introduced. This reduce the number of syllables in the teacher’s speech as perceived by DeeChee, using the Syllabifier software. Instead of collecting all possible syllables, the syllabifier processed the speech stream to exclude phoneme strings that were unlikely to be one-syllable words. The number of candidate strings reduced significantly, see Table 3, while the number of correct words perceived and reinforced by the teacher was close to that in set 1 (3 rather than 4). In contrast, other salient one-syllable words spoken by DeeChee were filtered out before they could be candidates for the teacher to notice. On average, 56% of salient words with canonical phonemic representation were excluded, and we decided not to use it again.

However, it indicated that, given a smaller candidate set of syllables, performance of the process with full reinforcement was of a similar standard to set 1. Results indicated that a filtering process could be worth investigating further.

Sets 3, 4 and 5 used the same program as set 1, but the guidelines given to participants differed. We tried to progressively lower the cognitive load so that the participant would focus on listening to DeeChee’s speech and detecting any words. Examples of the guidelines for the first and fifth set are given in Appendix S1.

One explanation of low scores on word learning is that a few teachers praised DeeChee whatever it said, in one case 10 times in the first 4 minutes, so non-words were erroneously reinforced.

### Evaluation of Word Learning in Set 1 to Set 5

We hypothesized that performance would improve from set 1 to set 5. To evaluate this we wanted to score each set for words correctly learnt (*true positives*) balanced against non-words learnt (*false positives*), both for simulated reinforcement and for real reinforcement. In the latter case we need to take into account *false negatives*: the words that DeeChee has produced but the teacher missed, or where the heuristic failed. In the case of simulated reinforcement there are by definition no false negatives.

We can look at *false positives* in two ways: first, we can take the “non-words” learnt in error, ignoring “other words” learnt, like “this” and “that”, proper but non-salient words. Secondly, we can take both “non-words” and “other words”.

**Figure 3:**
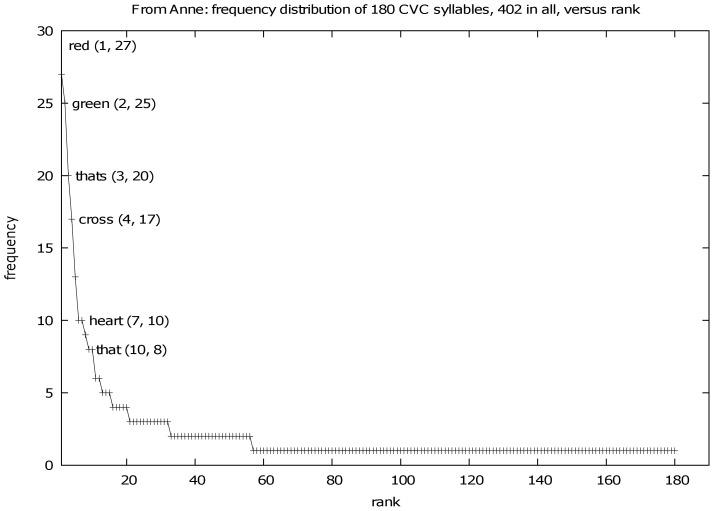
Zipfian relationship between frequency of CVC words and rank. Zipfian relationship between frequency of one-syllable CVC words in phonemic form, as perceived by the robot, and rank of the word. Recall that ‘C’ represents a consonant or a cluster of consonants, V represents a vowel. Example taken from participant 4A.

**Figure 4:**
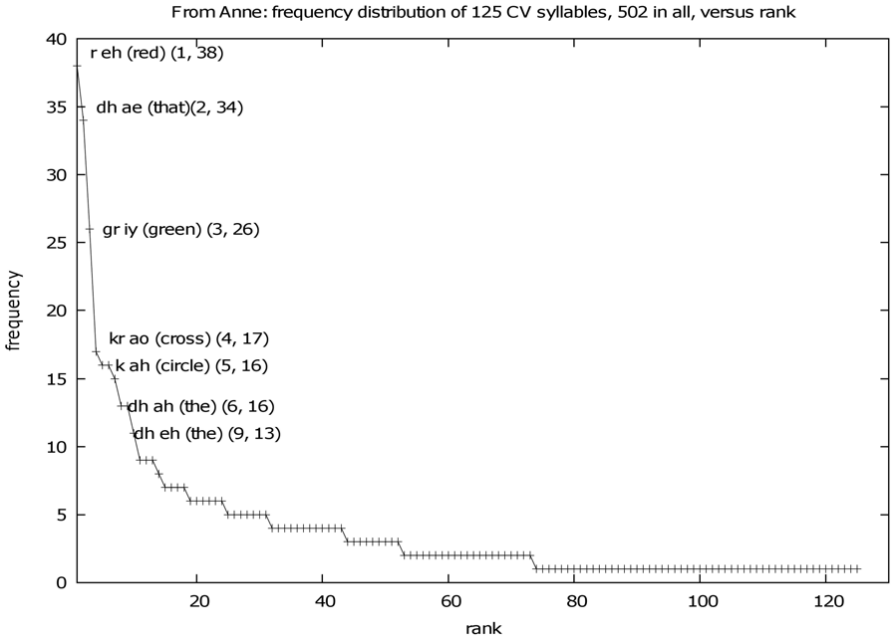
Zipfian relationship between frequency of CV syllables and rank. Zipfian relationship between frequency of CV syllables, in phonemic form, as perceived by the robot, and rank of the syllable. Example taken from participant 4A.

A standard method commonly used in natural language processing, is to derive the F-measure [61], [62]. This is appropriate for binary classification tasks on highly skewed distributions - see for example the typical distribution in Figures 3 and 4.

Let *t_p_* be *true positives*, *f_p_* be *false positives*, *f_n_* be *false negatives.*

Using standard terminology, *P* is Precision, *R* is Recall, *F* is the F-measure where
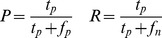
then



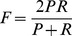
on the assumption that *P* and *R* are equally weighted. (The formula can be adapted to give more weight to one or the other.) The higher the F-measure the better the performance. If all *true positives* are learnt with no *false positives* or *false negatives* the F-measure will have a maximum value of 1.0.

Using this approach we can get an F-measure for each set under each of 2 conditions - real reinforcement and simulated, F1-1 and F1-2. We also repeated the analysis using the different definition of false positives: this second time we included the “other” words in the false positives, to give scores F2-1 and F2-2.

The results shown in Table 7 are discussed in Section Discussion below. A significant finding is the difference beween real and simulated reinforcement, the gap between what salient words were reinforced and what words could have been if they had been noticed by participants. The F-measures also show that there is a trade off between real salient words learnt and non-words erroneously learnt.

**Table 7:** F-measures.

Set number	F1-1	F1-2
	Real reinforcement	“Simulated reinforcement”
1	0.26	0.80
2	0.38	0.64
3	0.21	0.72
4	0.34	0.77
5	0.38	0.72
	**F2-1**	**F2-2**
1	0.25	0.77
2	0.31	0.53
3	0.18	0.62
4	0.32	0.71
5	0.33	0.65

F-measure for each set under each of 2 conditions: real reinforcement and “simulated reinforcement”, F1-1 and F1-2. “Simulated reinforcement” is based on the number of salient content words spoken by the robot. Most of these were not noticed and so not reinforced by the participant. The analysis is repeated using the different definition of false positives, to include the “other” words in the false positives, giving scores F2-1 and F2-2. “Other” words are proper words like “this” and “that” but not salient content words, the names of shapes and colours. See text.

There is an indication that word learning improved as the experimental programme progressed, but samples are too small for statistical significance.

### An Individual Case

It is illuminating to examine single cases where the participants interacted with DeeChee in a way that promoted learning. With participant 4A DeeChee had a high rate of phoneme recognition, and there was also effective dialogue leading to reinforcement. Three words were learnt, as well as three non-words. In this case no proper words were uttered by DeeChee but missed by 4A; an excerpt from her dialogue is shown above.

Table 8 shows the word frequencies in the top ranks, while the frequencies of the CVC syllables as perceived by DeeChee in dialogue with 4A are shown in Table 9.

**Table 8:** Word frequencies from orthographic transcriptions, participant 4A.

Rank	Word	Frequency
1	a	53
2	red	51
3	thats	33
4	green	32
5	you	26
6	and	24
7	cross	21
8	blue	21
9	heart	20
10	circle	17
11	we	15
12	that	14

Excerpt from ranked frequencies of words spoken by participant 4A. First 12 of 92 shown.

**Table 9:** Syllable frequencies perceived by robot DeeChee.

rank	CVC			CV		
	phonemic form	orthographic form	frequency	phonemic form	orthographic form	frequency
1	r eh d	red	27	r eh	part of red	51
2	gr iy n	green	25	dh ae*	part of that(s)	34
3	dh ae ts	thats	20	gr iy	part of green	26
4	kr ao s	cross	17	kr ao	part of cross	17
5	k ah l	part of circle	13	k ah	part of circle	16
6	r eh ds	part of red circle	10	dh ah*	the/that(s)	16
7	hh ah t	heart	10	t ah		15
8	s er k	part of circle	9	hh ah	part of heart	13
9	y eh s	yes	8	dh eh*	the/that(s)	13
10	dh ae t	that	8	bl uw	blue	11

Example from participant 4A.

Excerpts from ranked frequencies of CVC syllables spoken by participant 4A, as perceived by DeeChee. First 10 of 180 shown. Note the starred entries showing variable phonemic form for some function words. See graphical representations in Figures 3 and 4.

Of these the words *red, green, cross* were uttered by DeeChee, heard by 4A who responded with *well done, good* or *yes*. The heuristic selected the intended syllable from DeeChee’s previous multisyllable utterance which then passed into DeeChee’s lexicon. The relationship between the frequency of those syllables and their rank is shown in Figure 3. The distribution has a Zipfian character and the salient learnt words are among the highest ranking. The distribution of CV syllables has similar characteristics as shown in Figure 4.

## Discussion

DeeChee successfully acquires some salient one-syllable word forms in real-time through unconstrained embodied linguistic interactions with naive participants.

An experiment lasting just 8 minutes cannot compare with a child’s experience, immersed in a linguistic environment. However, some experiments with infants, learning the statistical distribution of phonemes, report results after just 2 minutes [25]. An appropriate analogy for a robot language learning experiment is with a situation where a carer is explicitly aiming to teach a child, for instance in a therapeutic setting.

We wanted to explore human-robot interaction and were deliberately not prescriptive. However, leaving participants to talk naturally opened up possibilities of a wide range of behaviour, possibilities that were certainly realized. Some participants were better teachers than others: some of the less good produced very sparse utterances, while other talkative participants praised DeeChee whatever it did, which skewed the learning process towards non-words.

A factor that affected the results was the level of phoneme recognition through the SAPI 5.4 recognizer. This may have been exacerbated by the unavoidable use of noisy fans in the small room with the robot where experiments took place.

Turn taking was implemented on a timed basis and some participants over ran their turn, speaking at the same time as DeeChee, in which case a small amount of the participant’s speech was not perceived by the robot. Thus the statistics for words spoken by the teacher and syllables perceived by the robot need to be interpreted with this in mind (Table 3).

The overall level of performance was much lower with real-time human reinforced learning than with simulated reinforcement against a stored lexicon. This is shown by the F-measures in Table 7. We can see some indications of learning performance, but samples are too small to be statistically significant. Specific points to note are that the performance of set 2 was affected by the syllabifier excluding many valid words. The F1-1 and F2-1 measures were not out of line with those of the other sets, but the scope for improvement was limited by the exclusion of many candidate words uttered but not recognized, as shown in Table 5 and indicated by the F1-2 and F2-2 measures. However, it did suggest that by reducing the choice cognitive load declines and performance improves.

The poor performance of set 3 could be partly attributed to the Guidelines: the explicit instruction to make an approving comment if DeeChee uttered a proper word was left out, leaving the participant to act spontaneously without setting up any expectation of reinforcement. This set also had the lowest phoneme recognition rate.

The performance with sets 4 and 5 is marginally improved (see Tables 6 and 7), but the samples are too small to be statistically significant.

By looking at group results the variability in performance means that some interesting results from effective participants are masked. We suggest that it can be more informative to examine individual cases, as above. This is analogous to some research practices in child language and developmental neurocognition research, where children are selected for investigation because they display the characteristics in which the researcher is interested while others do not [63] (page 412).

### Why it Works

We have shown that in some cases the robot was able to bootstrap the learning of some word forms through interaction with a naive participant. This indicates that a mechanism like the one described here could be a contributory factor in the acquisition of word forms.

The first reason that words were learnt is that they were, as expected, repeatedly spoken by the teacher, as illustrated in Table 8.

A second reason is that non-salient word strings are typically quite variable, so that their frequencies are spread about. This observed phenomenon is the basis of a number of automated plagiarism detectors, where precise matches of short lexical strings indicate copying, e.g [64].

A third reason is that the phonemic representation of speech from the teacher to DeeChee is not a uniformly stable mapping of sounds to canonical phonemic word forms, as illustrated in Table 9. The frequencies of syllables in words with variable phonemic forms may be attenuated compared with those in salient content words, or parts of such words. It has long been realized that there is in practice a great deal of variation in spontaneous speech, as described by Greenberg in an analysis based on the Switchbord corpus [19]. One example of his findings is that the word “and” is represented phonetically in 80 different ways in 4 hours of manually annotated spontaneous telephone speech (*ibid* page 163).

It is worth comparing results from the Switchboard (*ibid* page 169) and TIMIT corpora [65]. The latter is also derived from spontaneous telephonic speech, but in this case the speech is transcribed and then read. The phonetic realization of words is found to be closer to their canonical form in the read TIMIT material than in the case of Switchboard which is taken directly from the original speakers. For CVC syllables (recall that “C” is either a consonant or a cluster of consonants in our notation) the onset is usually realized in canonical form for both corpora, but the nucleus and, more particularly, the coda are realized in more variable ways in the Switchboard corpus.

However, the variability in pronounciation of words in spontaneous spoken language, which at first appears a problem, may in fact contribute to the learning of early word forms. This is because salient content words are more likely to have a consistent canonical phonemic representation than function words, thus their frequency builds up and so does their consequent influence on the learner’s utterances.

Words of high information valence (these are typically infrequently occurring referential constituents of a nominal phrase [i.e., nouns or adjectives]) tend to be pronounced in canonical fashion, while common lexical items, particularly pronouns, conjunctions and articles, generally depart from canonical form with regularity. [19] (page 172, brackets in the original).

This was also noted by Pierrehumbert and Hirschberg, commenting on the observation that stress in speech sounds is related to information carried, and also that.

Syllables with greater stress are more fully articulated than syllables with less stress [66] (page 272).

The information valence of words affects not only their prosodic characteristics, but also their phonological realization. Function words are often more common in orthographic form, as are syllables bridging words that include a function word (such as *s ih z* from *this is*, the Sandhi effect). However, their phonological form may vary, and the frequency of salient content words as perceived sounds may be just as significant. Since it has been well established that infants are sensitive to the distributional statistics of sounds they hear [25], [67] the frequency of phonemically represented content words may play a role in word form acquisition as it does in our model, as Figure 3 illustrates. See also that the frequencies of CV syllables in Figure 4 include parts of these salient words at the top of the range, among orthographically frequent function words.

Research in child language acquisition has not fully assimilated the facts concerning phonological variability. Though acoustic variation has sometimes been recognized, and attempts made to address the issue [68], [69], this is not always the case. In some received work canonical representation is assumed: Child Directed Speech (CDS) is transcribed orthographically into words, which are then represented phonemically by looking up entries in a dictionary. One example is the well known Brent corpus [70]. In a recent collection of articles on computational models of child language learning MacWhinney cites four authors who use this corpus to evaluate their models [71] (page 478). Research in the field has not ignored this problem, and various approaches have been taken to amend the orthographic transcript. For instance, after words are replaced by phonemic forms, using an on-line dictionary, these forms are input to a set of rewrite rules that introduce phonological alternations into the string, such as assimilation and vowel reduction [72], but such approaches do not fully compensate for the loss of information. Some special purpose lexica have been developed with entries for the most common phonological variations, but though they can produce modest improvements these are reported as not matching the performance of the human listener [19].

Words that carry little information are more likely to have variable phonetic representation, so increments in their frequencies are spread about and “diluted”. On the other hand information bearing words are more likely to have consistent canonical forms, so their frequency builds up and they consequently have a significant influence on the productions of the learner. Salient words can emerge as more frequent sounds. The variable phonetic representation of spontaneous spoken language and its phonemic realization, which at first appears a problem, may in fact contribute to the learning of early word forms. We are back to the injunction of the philosopher Wittgenstein: “Don’t think but look! … the more we examine actual language, the sharper becomes the conflict between it and our requirement […] the crystalline purity of logic” [73] (sections 66 and 107).

### The Wider Context of Human Robot Interaction (HRI) and Future Work

This work contributes a thread to the wider context of developmental robotics. It is in line with the tenets of a programme scaffolding linguistic learning on individual and social learning [74] (page 188) in that:

- it does not require substantial innate hardwiring - sensitivity to frequencies of sounds is the key motivator that enables learning
- it is grounded in recurrent patterns of embodied experience and social interactions. The problems associated with real, naive participants interacting contingently with a robot could be avoided by having off-line experiments and trained participants. However, this would mean obscuring the real world environment that we want to investigate.

The process described here precedes the acquisition of sophisticated cognitive capabilities and the ability to analyse more highly structured linguistic input. But it feeds into these higher level functions by contributing to the detection of salient terms, and hence to the wider field of associating meaning and usage with word forms [3]. In the immediate future the next step is to investigate how different factors in salience detection are correlated and can be integrated, in particular with prosodic information. Prosodically marked up speech data from subsequent experiments in similar scenarios is available and awaiting analysis.

Our work demonstrates a HRI platform in which it is possible to sustain interaction to achieve rudimentary word form acquisition in real-time using a simple frequency dependent probabilistic generation mechanism, together with human reinforcement. This work shows the potential of human-interaction systems to be used in studies of language acquisition, and the iterative development methodology highlights how the embodied nature of interaction may bring to light important factors in the dynamics of language acquisition that would otherwise not occur to modellers.

## Supporting Information

Appendix S1 **Guidelines given to participants.**
(PDF)

Video S1 **Example of a dialogue between robot and participant.**
(WMV)
