# Supplementary material for: Interactive Language Learning by Robots: The Transition from Babbling to Word Forms
Source: PLoS One. 2012 Jun 13;7(6):e38236. doi: 10.1371/journal.pone.0038236 (PMC3374830; doi:10.1371/journal.pone.0038236)
Supplement: Appendix S1 — Guidelines given to participants. (PDF) [file pone.0038236.s001.pdf]

# Appendix S1: Guidelines given to participants

## Guidelines for Set 1

**Experiments with robot DeeChee**

In these experiments we are investigating how language might be learnt by robots.

We simulate conversations that might take place between adult carers and small children. In this case the small child is replaced by a small robot.

Today you are asked to talk to our robot DeeChee, as if it is a small child. DeeChee will start babbling, then you try and teach the names of shapes and colours.

**Instructions**

- You will have a microphone on a speaker headset. You have boxes with coloured patterns on them.
- Your job is to talk with DeeChee, teaching it the names of shapes and colours. To begin with DeeChee can only babble: it does not know any proper words.
- You take turns talking to each other. When DeeChee is listening to you it will smile.
- But WATCH! When DeeChee blinks and stops smiling it is about to talk. Please listen carefully to DeeChee babbling, even if it sounds nonsense!
- If it says a proper word, even if it is not the right word, please make an encouraging comment!

You will be told when to start and finish the session.

There are two sessions of about 4 minutes each.

## Guidelines for Set 5

**Experiments with robot DeeChee**

[First part shown to participants in advance, as introductory material, before simplified "Instructions" below]

In these experiments we are investigating how language might be learnt by robots.

Today you are asked to talk to our robot DeeChee, as if it is a small child. You take turns talking to each other.

- You will have a microphone on a speaker headset. You have boxes with coloured patterns on them to show to DeeChee.
- DeeChee will start babbling, but does not know any proper words. You try and teach the names of shapes and colours.
- There are two sessions of about 4 minutes each.
- You will be told when to start and finish the session .

**Instructions**

Please listen carefully to DeeChee babbling. If it says a proper word, even if it is not the right word, please make an encouraging comment!
